# Surgery

**Published:** 1891-10

**Authors:** John Parmenter


### Progress in Medical Science.

### SURGERY.

CONDUCTED BY

JOHN PARMENTER, M. D.

### ETHER SPRAY AND COCAINE.

SCHLEICH (*Deutsche Medicinal Zeitung*, 1891, *No.* 44,) recommends the previous spraying with ether of any part in which cocaine is to be used. It must last half a minute, after which time one can employ numerous injections of cocaine—not more than 0, 01-0, 03—cocaine being necessary to make large areas completely anesthetic. This same method is to be employed in cases where division of deeper layers is necessary, each one being treated in turn, as was the skin. In this way the author has made small resections and bone operations without giving any pain.

### DESTRUCTIVE ACTION OF CARBOLIC ACID.

A. FRANKENBURGER (*Inaug.-Diss.*, *Erlangen*, 1891,) warns against the injury to skin, and even bone, which may result from the long-continued application of weak (3, 2½, and even 2 per cent.) carbolized applications, especially upon peripheral portions of the body, such as the fingers. This effect is due in a small measure to the action of carbolic acid upon the vaso-motor system, but in the main to its destructive effect upon the red and white blood corpuscles. This induces, partly in a mechanical and partly in a chemical way, stasis, first in the capillaries, and, if the action of the drug be continued, then in the larger veins and arteries, with the result that the nutrition of the part is interfered with, and the removal of harmful substances hindered. The maceration of the epidermis caused by the acid favors evaporation, so that the gangrene is a dry one, a mummification as it were. The author believes that even weak carbolic solutions as external applications are to be either completely discarded, or to be used only with the very strictest precautions, and that the sale of carbolic water by druggists should be wholly stopped.

### INDICATIONS FOR TREPHINING.

DEAVER (*Annals of Surgery*) gives the following indications for trephining :

1. Simple depressed fracture with or without brain symptoms.

2. Compound depressed fracture with or without brain symptoms.

3. Impacted fracture, simple or compound, with or without brain symptoms.

4. Comminuted fracture, simple or compound, with or without brain symptoms.

5. Compound fissured fracture, with depression of bone, without brain symptoms.

6. Compound fissured fractures, with depression of bone, with brain symptoms.

7. Compound fissured fractures, without depression of bone, and without brain symptoms, in which there is bleeding through the fissure or fissures.

8. All punctured, incised, and gunshot fractures.

---

### TREATMENT OF PENETRATING WOUNDS OF THE ABDOMEN.

Dr. Lanphear (*Medic. Review—Internat. Journ. of Surgery*, 1891, —*July*) makes the following conclusions regarding the treatment of penetrating wounds of the abdomen :

1. All cases of penetrating gunshot wounds of the abdomen demand laparatomy ; most others also require it.

2. The operation should be done immediately after the injury, if possible, so as to control bleeding before the patient is exhausted.

3. Any time within twelve hours may be regarded as the time of selection, but the lapse of many hours, or even days, need not prevent operation, since death from septicemia is likely to occur.

4. A condition of collapse is not an insurmountable contraindication.

5. The existence of peritonitis demands, rather than forbids, an operation.

6. In gunshot wounds, Senn's hydrogen test should not be employed, as the indications are *always to operate* ; perforation of the intestines is not necessary to render the wound fatal. In other penetrating wounds the test may be employed.

7. Laparatomy is, in such cases, comparatively an insignificant operation. Any surgeon of ordinary skill ought to be able to successfully operate.

8. In case of emergency, the operation here described can be made without an elaborate set of instruments. A success can be obtained by the use of only: (*a*) a knife; (*b*) scissors; (*c*) needle and thread; (*d*) hemostatics; and (*e*) *good judgment.*

---

### TREATMENT OF BURNS.

MONS. GRIGORESEN (*Bulletin de la Societé de Biolojie—Internat. Jour. of Surgery, August*, 1891,) makes the following remarks on the treatment of burns: Pure glycerine applied to the skin in the case of burns acts as an anesthetic, especially when applied early. In severe cases two or three applications should be made, so that the parts are kept constantly wet with glycerine. At first a slight burning feeling is experienced, which soon gives place to a local anesthesia somewhat resembling that produced by carbolic acid. Under this treatment the inflammation is subdued almost completely, and only a slight cicatrix is usually felt. The glycerine should be slightly rubbed in, and the parts then covered with a protective dressing.

---

### PREVENTION OF RUST ON SURGICAL NEEDLES.

DR. DAWBARN (*N. Y. Med. Journal*) makes the following suggestions regarding the prevention of rust on surgical needles. He states that dry sterilizing of needles at the usual temperature is apt to injure their temper and cause subsequent bending during use; while boiling or steaming induces flecks of rust, and glycerine has failed. He recommends keeping the needles in alcohol after they have been treated in the following manner: The needles are immersed in benzine to remove grease, then running them through a towel the points are plunged into a piece of cork the size of a pea —to avoid dulling from jotting—and finally with their corks are placed in a wide-mouthed, glass-stoppered bottle filled with absolute alcohol. After using the needles, pass them through a thick, wet, soapy towel repeatedly, cleanse the eye with a thread, immerse in benzine, and finally replace in the alcohol. The latter substance is an efficient disinfectant, besides being an excellent protector against rust.

---

### EFFECT OF OPERATIONS PER SE.

J. WILLIAM WHITE (*Annals of Surgery*, August and September, 1891,) in an extended article on the curative effect of operations *per se*, sums up with the following conclusions:

1. There are large numbers of cases of different grades of severity and varying character, which *seem* to be benefited by operation alone, some of them by almost any operation.

2. These cases include chiefly epilepsy, certain abdominal tumors, and peritoneal effusions and tubercle, though the improvement in the latter is, perhaps, to be explained on general principles.

3. Of the possible factors, which, by reason of their constancy, must be considered. Anesthesia seems least likely to have been effective. The other three, viz., psychical influence, relief of tension, reflex action may enter in varying degrees into the therapeutics of these cases, and taken together serve to render the occurrence of occasional cures less mysterious.

4. The theory of accident or coincidence scarcely explains the facts satisfactorily.

---

### PISTOL-SHOT WOUNDS OF THE SKULL.

Bradford (*Boston Medical and Surgical Journal*, June 11, 1891,) in an article on pistol-shot wounds of the skull, makes the following *résumé:*

In penetrating pistol-shot wounds of the skull, death is to be anticipated in a large majority of cases. Recovery, however, does occur in a certain number of instances. The treatment should be the treatment of a penetrating fracture of the skull (that is, a fracture from any sharp instrument, a nail or spike). The scalp should be dissected away from the injury, and the bone thoroughly examined. Loose fragments of bone should be removed. A trephine should be used if it is considered necessary to thoroughly cleanse the wound, or to give egress to any collection of fluid or blood that may be below the dura. If the dura is penetrated, and if there is any bulging of the dura, the dural opening should be enlarged. The brain substance should be thoroughly irrigated and washed, but not probed or treated with violence. It is unnecessary to state that the strictest asepsis is desirable.

---

According to a recent visitor to Japan, the people of that country pay more attention to personal cleanliness than any other people in the world. High and low bathe all over at least once a day, and sometimes oftener.—*College and Clinical Record.*

